# Interactive Effects and Mediating Roles of Multiple Factors That Influence Learning Adaptative Growth of International Students: Evidence from China

**DOI:** 10.3390/bs13080682

**Published:** 2023-08-14

**Authors:** Jian Li, Eryong Xue, Yukai Wei, Yiling Guo

**Affiliations:** China Institute of Education Policy, Faculty of Education, Beijing Normal University, Beijing 100875, China; jianli209@bnu.edu.cn (J.L.); yukai.wei@student.unimelb.edu.au (Y.W.);

**Keywords:** learning adaptability, international students, learning performance, international education in China

## Abstract

The learning adaptability of international students is pivotal to the success of sustainable international higher education development. The purpose of this study was to explore what factors affect the learning adaptability of international students in China through structural equation modelling and mediation analysis. The data collected through a questionnaire from the overseas students were analysed, and the reliability and validity were also tested. The findings show that the influencing factors that affect learning adaptability of international students in China comprise seven variables: learning attitude, motivation to study abroad, learning ability, language proficiency, learning environment, teaching management and social relations. In addition, when language proficiency is used as the mediating variable, the motivation to study abroad has a significant positive impact on learning attitudes, with an influence coefficient of 0.185 and an effect proportion of 35%, which is a partial mediator. When social relationships are used as the mediating variable, study abroad motivation has a significant positive impact on learning attitude, with an influence coefficient of 0.058, which is completely mediating.

## 1. Introduction

International students’ learning adaptability is significant to the quality and sustainable development of international higher education. International higher education has rapidly developed in recent years [1]. As a result, the number and scale of international students have also gradually expanded. An increasing number of international students wish to study in China. Conceptually, “international students” generally refers to students studying or conducting research abroad, and specifically refers to foreigners studying or conducting research in China [2]. By 2022, the number of international students in China had exceeded 440,000, making China a popular destination for foreign students. The learning adaptability of overseas students is reflected in the process of gradually adapting to the new learning environment and cultural differences [3]. As the object of study in this research project, international students need to undergo a series of adaptation processes to adapt to the culture, learning and living environment [4]. With the deepening of cultural exchanges and clashes worldwide, scholars are paying increasing cross-regional attention to different regions and cultural groups. For example, research focuses mainly on cross-cultural adaptation, which also includes overseas students’ learning adaptation. It is believed that the quality of education services for overseas students is directly related to the impression of, and knowledge about, China by students worldwide, as well as the friendly relations between China and other countries. Therefore, we should pay attention to improving the quality of educational services. In the context of higher education, international students need to pay attention to the establishment and implementation of educational programs. Higher education should offer a wide range of geographical, sociocultural, educational and other courses. Universities should provide international students with opportunities to participate in various courses to help them participate more effectively in cross-cultural learning [5].

Few studies focus, however, on the interactive effects and mediating roles of the multiple factors that influence the learning adaptative growth of international students. Therefore, this study aims to explore what factors affect the learning adaptability of international students in China using structural equation modelling and mediation analysis. The research questions include the following:

1. What factors affect the learning adaptability of international students in China?
2. What is the influencing role of language proficiency in motivating study abroad and learning attitudes?
3. What is the role of social relationships in study abroad motivation and learning attitude?

### 1.1. Learning Adaptability and International Students’ Cross-Cultural Ability

The concept of learning adaptability has not been uniformly defined academically. The earliest concept of learning adjustment comes from Tinto (1975), who introduced the concept of “academic system” when studying the long-term model of college dropouts. The concept refers to a system of academic factors, including academic performance and intellectual development [6]. Students’ learning adaptability is closely related to learning motivation, goals, environment satisfaction and effectiveness. For international students, learning adaptation refers to the process of constantly adjusting their psychology and thinking patterns in a new learning environment to overcome confusion and reach their academic goals [7]. For example, adaptive learning is defined as a type of ability in the process of learning, which allows an individual to actively adjust learning motivation and behaviour according to changes in the internal and external learning environment and his or her learning needs, improve learning ability, and coordinate psychology and behaviour. Learning adaptability refers to a psychological tendency of learners to overcome difficulties in the learning process to maintain harmony with the learning environment and finally achieve good learning results [8]. Learning adaptability is reflected not only in learners’ simple and passive “adaptation” to the teaching method and learning environment, but also in the whole learning process. In other words, learners can achieve more efficient and lasting learning outcomes by improving their learning ability. Adaptability means that students can adjust their learning state independently according to their attitudes and environment changes to achieve a balanced development state between the internal and external learning environment. Learning adaptation means that when students are faced with the learning environment and needs, they can actively adjust themselves to achieve psychological and behavioural balance. This process mainly includes five aspects: learning motivation, learning ability, environmental factors, teaching method and learning attitude [9].

### 1.2. Current Studies on Learning Adaptability of International Students

Learning adaptation refers to actively adjusting oneself to achieve a balance between psychology and behaviour to adapt to the environment and learning needs. Using a questionnaire method, Wei and Li [10] found that college students’ learning adaptability mainly includes five aspects: learning motivation, learning ability, environmental factors, teaching mode and learning attitude. Zhu et al. [11] conducted an open questionnaire survey of experts, teachers, and students, used the two dimensions of learning motivation and learning behaviour, and constructed the structure and components of college students’ learning adaptability through eight factors: self-management, method application, professional interest, pressure regulation, hell-seeking tendency, information utilisation, environmental choice and knowledge transformation [12]. Yang et al. [13] conducted a study of 918 college students from four different universities and performed exploratory factor analysis and confirmatory factor analysis on the survey data. The results showed that the main factors of college students’ learning adaptation include learning motivation, teaching mode, learning ability, learning attitude and environmental factors. The five factors extracted from the scale fit well with the imagined model, and the test was found to have high reliability and validity. Tian and Lu [14] found that there was a significant positive correlation between the learning motivation of international students in China and their learning adaptation [15]. These findings are of great significance for improving the learning effect and adaptability of overseas students. By investigating foreign students in China, Li et al. [16] found that foreign students have poor language foundation and lack of enthusiasm for learning. Additionally, they considered the school curriculum to be unreasonable, and yet were generally satisfied with school infrastructure [17].

### 1.3. Influencing Factors of Learning Adaptation for International Students

The factors that affect the learning adaptability of international students can be divided into internal factors and external factors [18,19,20,21]. Li et al. [16] found that effective learning becomes more difficult when there is an essential difference between the teaching methods that cross-cultural learners are accustomed to and the new teaching methods that they face. During cross-cultural learning, learners may face the problem of a lack of knowledge and experience, resulting in the inability to effectively construct knowledge. At the same time, effective communication is lacking, which affects the quality of learning. Through interviews with Chinese students in the United States, the main cross-cultural learning adaptation problems of overseas students were found to be differences in learning methods, the strength of students’ self-management ability and the lack of communication skills. Differences among international students in cross-cultural learning adaptation were shown to vary by nationality, gender, length of stay in China and language level [22]. At the same time, students have adaptation problems in language learning, understanding classroom content, teaching methods and curriculum assessment. In the study of the influencing factors of overseas students, language level has been considered very important by most scholars. A study of the cross-cultural learning adaptation of central Asian students in China found that they were relatively satisfied with the campus environment and school facilities, whereas their learning adaptation difficulties were reflected mainly at the language level. A study of students in four universities found that, compared with students studying in Chinese, students studying in English have stronger learning adaptability and there are significant differences between the two, especially when studying language and literature courses, with learning in Chinese being more difficult. The survey also concluded that the main reason why international students choose China as a study destination is to learn Chinese. Compared with foreign students from other countries, international students in China pay more attention to language learning. It was found that teaching assessment forms, educational management and other factors play important roles in the cross-cultural learning adaptation of overseas students [14].

By exploring the factors that influence foreign students’ adaptation to learning, scholars have also put forward corresponding strategies to solve the problem of cross-cultural learning adaptation, which can be roughly divided into the following aspects. International students should actively cultivate cross-cultural active communication abilities, strengthen cultural empathy, improve knowledge of the country where they study and develop the mentality to better adapt to and integrate into the local cultural environment. At the school level, scholars have proposed requirements for teachers and administrators, respectively [13]. For example, Cao et al. [3] concluded that, in terms of intercultural education, the development of intimacy, sincerity, tolerance, mutual respect, mutual understanding and mutual solidarity by teachers and students can promote cross-cultural communication. Establishing counselling programmes for international students by schools provides direct psychological intervention for international students to enhance their life in China and their Chinese communication ability, improve the school’s social support network and strengthen the teaching of Chinese culture, improving the cross-cultural adaptability of international students. At the social level, Gong et al. [6] in an empirical study on cross-cultural adaptation of overseas students in Beijing, proposed promoting the convergent management of Chinese and foreign students and Chinese culture, stimulating learning interests, strengthening research on target groups and establishing effective channels of cultural transmission to solve leading problems and contribute to building a liveable world city [23].

### 1.4. Theoretical Perspective: Cross-Cultural Adaptation Theory

Kim and Kim [9] developed a model that aims to describe the interaction of multiple factors that influence cross-cultural adaptation growth, to reflect the dynamic process of cross-cultural adaptation. The cross-cultural adaptation theory is a spiral model. When foreigners enter a new environment, they face conflicts with the culture of the host country. Some people may choose to avoid problems, while others will try to learn the culture of the host country to seek solutions to the conflicts. The factors that influence such growth mainly include communication, environmental and personal characteristics.

Communication is the exchange of ideas and feelings between people and groups, aiming at achieving consistency of ideas and a smooth flow of emotions. Communication needs a platform. Foreigners often form groups with cultural values different from those of the host country, which is also a factor in communication, namely, interpersonal transmission between natives. In contrast, in the host country, interpersonal communication is a platform that provides emotional, information and social support. The main forms of mass communication are newspapers and network platforms in host countries. Communication and interpersonal communication in the host country are very important for foreigners who have not mastered interpersonal language communication. Here, the host country provides a platform for foreigners. They can discuss their cross-cultural adaptation problems and share their ideas on how to solve them. Those who arrived in the host country earlier can share their experiences to help other foreigners.

**Figure 1:**
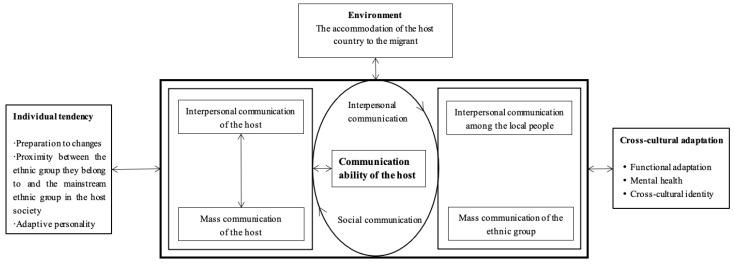
Model of acculturation process (Adapted from [9]).

Environmental factors generally include the host country’s acceptance of migrants, conformity pressure and power of the stranger population. The host country’s acceptance of migrants refers to the extent to which the host society helps foreigners to build in-country interpersonal networks. For example, if there is a significant difference between the culture of the host country and that of the foreigner’s country of origin, the foreigner must learn and observe the cultural customs of the host country. Every foreigner needs to adjust to a new psychological and emotional state after moving to a new society. It often depends on how long they have lived abroad and other factors. Some expats are emotionally determined to learn and successfully adapt to a new society, while others may be reluctant to adapt to local life for psychological reasons, such as the feeling that they have been deprived of their original cultural identity. Before going abroad, foreigners’ study of the language and culture of the destination country helps them adapt better because they are psychologically prepared to deal with new challenges while learning about the new culture. For those whose culture of origin is more like that of the host country, there is less cross-cultural adaptation pressure experienced when adapting to the new culture, and vice versa (see Figure 1).

## 2. Materials and Methods

### 2.1. Sampling and Data Collection

Taking international students at S University as the investigation object, this study examines the problems experienced with the learning adaptability of international students in China. The research objects of this paper are 4660 overseas students studying at the undergraduate level at B University, of whom 2400 are female and 2260 are male students. The age of the subjects was between 19 and 23 years old. The surveyed areas or origin are mainly in Asia; thus, most of the research participants have a high level of Chinese proficiency. Among the subjects, 186 have passed HSK6. The funds for their study abroad come mainly from scholarships, and many subjects have obtained full scholarships.

### 2.2. Measurement and Questionnaire Design

We designed a questionnaire survey based on Richter’s five-level scale and applied it to 4660 foreign students in China. Through this method, we collected corresponding data for analysis to explore the influencing factors of learning adaptation of overseas students in China. According to the research objectives, the questionnaire focuses mainly on overseas students at S University. We aim to understand the learning adaptability of international students at S University; to compare and analyse the differences in the learning adaptability of different genders, different language levels and different regions of origin; and to summarise the learning adaptability problems of international students in China. The objective is to make suggestions for improving the learning adaptability of international students in China.

The questionnaire comprises two parts. The first part contains 13 questions to investigate the situation of international students at S University, including personal information, Chinese level, and other basic information. The second part is the learning adaptability scale for international students in China, which includes the individual, environmental and interactive dimensions. The individual dimension entails learning attitude, study motivation, learning ability and language level. The environmental level includes the learning environment and teaching management. The interactive dimension includes social relationships. A Likert five-point scale was used to design the questionnaire (false, mostly false, unsure or don’t know, mostly true and completely true). According to the scoring method, each level of the forward statement questions is scored from 1 to 5, while the reverse statement questions are scored from 5 to 1. By adding the scores of each question and dividing by the total number of questions, the average score of the degree of learning adaptability of international students can be obtained. This average score is positively correlated with learning adaptability, meaning as the score increases, the adaptability improves.

### 2.3. Reliability and Validity Test of Questionnaire

In this paper, SPSS26.0 is used to analyse the reliability of the questionnaire content. The overall reliability and internal consistency of each dimension of the questionnaire are analysed. The final analysis results are shown in the following table. A value greater than Cronbach’s alpha coefficient of 0.8 indicates good reliability. The test analysis shows that the reliability of the learning adaptability questionnaire used in this paper is 0.937, indicating high reliability. At the same time, the reliability of each dimension is higher than 0.7, indicating a high level of reliability.

The table shows that, in terms of gender distribution, male students account for 51.50% of the sample, whereas female students account for 48.50%. Regarding age distribution, most individuals in the sample were 19–23 years old (2640 people, accounting for 56.65%), and 43.35% of them were over 23 years old. From the perspective of the distribution of international students, more than 40% of the students are from Asia, with 2030 respondents, whereas European students account for at least 13.95%, with 650 students. Regarding the amount of time studying Chinese, most of the respondents had studied Chinese for 12–24 months, with a total of 1850 people accounting for 39.70%. In total, 290 international students had studied Chinese for 1–6 months, accounting for 6.22%, whereas 660 foreign students had studied Chinese for more than 36 months in the receiving country, accounting for 14.16%. In terms of Chinese proficiency, 1860 international students passed the Chinese Level 6 test, accounting for 39.91%; 27 passed the Chinese Level 3 test, accounting for 5.79%; and 720 people did not take the Chinese proficiency test, accounting for 15.45%. Regarding scholarship distribution, 206 people received a full scholarship, accounting for the highest proportion, 44.21%. Among the respondents, 3770 came to China for the first time, accounting for 80.90%, while 287 students had never been abroad, accounting for 61.59%. Regarding understanding B University, 1640 students reported having an “understanding” attitude, accounting for the highest proportion at 35.19%. The number of people who did not know B University very well was at least 25, accounting for 5.36%; 81 reported knowing it very well, accounting for 17.38%. In terms of communication with people from different cultural backgrounds, 78 students had never engaged in cross-cultural communication, accounting for the smallest proportion of students at 16.74%, whereas 2320 people reported rarely participating in communication, accounting for the highest proportion (49.79%). Among all the respondents, 3360 had Chinese teachers for learning the Chinese language, accounting for 72.1% (see Table 1).

In terms of the specific indicators of relative quantification, the average values of Chinese learning time, Chinese proficiency and school understanding by international students in China are 3.056, 3.727 and 3.464, respectively, indicating that most of the students had spent some time learning Chinese (12–24 months), had reached a proficiency level of Chinese 4 and had a general understanding of the school (see Table 2).

As mentioned above, on the basis of the literature review, the questionnaire included seven factors affecting the learning adaptability of international students in China: learning attitude, motivation to study abroad, learning ability, language level, learning environment, teaching management and social relations, numbered as variables 1–7. In addition, structural equation modelling (SEM) was used to explore the mutual influence and functional relationship of the influencing factors. Among the factors that reflect learning adaptability, learning attitude, motivation, learning ability and language level are internal factors, whereas learning environment, teaching management and social relations reflect external factors. According to the literature review, learning attitude is a direct manifestation of adaptation effectiveness, and language level and social relationship constitute relatively core content among internal and external factors, respectively. Therefore, we explore the mutual influence and relationship between these three factors and determine the degree of influence and effect between variables.

## 3. Results

### 3.1. Model Validation Analysis

To further test whether the original model is valid, data fitting, model revision and model testing are carried out. The model fitting indexes are as follows: χ^2^/df is the chi-square degree of freedom ratio, GFI is the fit index, also known as the goodness of fit index, RMSEA is the root mean square of approximate error, CFI is the comparative fit index and IFI is the value-added fit index (χ^2^/df = 2.267, GFI = 0.963, RMSEA = 0.077, CFI = 0.914, NFI = 0.912, IFI = 0.914). The chi-square degree of freedom ratio is used to compare the fit degree between models and is a relative core judgment index. As the chi-square freedom ratio decreases, the degree of model fit increases. When the chi-square freedom ratio is less than 3, the model’s fit is ideal. The values of CFI, IFI and GFI are all between 0 and 1, and the closer the value is to 1, the better the model fit. Generally, values above 0.8 are acceptable and above 0.9 are excellent.

The main fit indicators all meet the judgment criteria, and the model is considered to fit well with the actual observation data. The established model of parental attitude influencing the effect of science education is a good theoretical hypothesis model. Structural equation models provide a quantitative way to understand the effects of and interrelationships between variables. These regression coefficients can be used to explain causality and relative influence in the model (see Table 3).

### 3.2. Path Analysis

Learning motivation has a significant positive impact on language proficiency; that is, improved learning motivation is positively correlated with the improvement of language proficiency. As students become more motivated, their Chinese proficiency increases. The standardised regression coefficient is 0.183, indicating the relative influence in the model. Learning motivation has a significant positive impact on social relations, and the standardised regression coefficient is 0.174, indicating that, as the international students’ learning motivation increases, their motivation composition becomes more reasonable and their ability to construct good social relations improves.

Learning ability has a significant positive impact on language level, and improvement in learning ability is positively correlated with improved language level. The standardised regression coefficient of 0.200 indicates the model’s relative influence. The standardised regression coefficient is 0.254, meaning that the improvement in learning ability is positively correlated with the improvement of social relations. The stronger the learning ability, the more able one is to build friendly and harmonious social relations.

Language level has a significant positive impact on learning attitude, and the standardised regression coefficient is 0.152; that is, the improvement in language level is positively correlated with improved learning attitudes. Students with higher language levels often have a more positive and appropriate attitude towards learning. The standardised regression coefficient is 0.290, meaning the improvement in learning environment is positively correlated with better social relations. A more favourable learning environment for international students can help them build good social relations.

**Figure 2:**
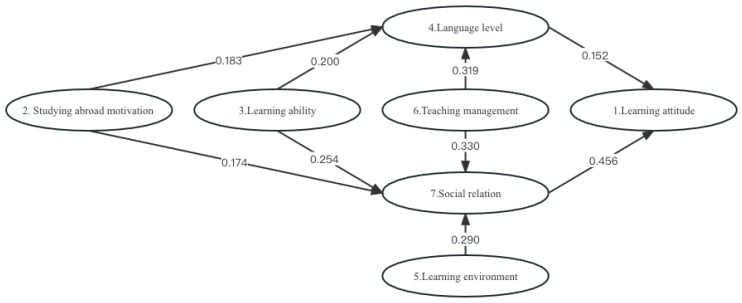
Path analysis of structural equation modelling.

Teaching management has a significant positive impact on language level; the standardised regression coefficient of 0.319 indicates that the improvement in teaching management is positively correlated with improved language level. The more scientific and reasonable the content of teaching management, the more conducive to improving the Chinese language level of international students in China. Teaching management has a significant positive impact on social relations, and the standardised regression coefficient is 0.330. The improvement in teaching management is positively correlated with improved social relations. Scientific and reasonable teaching management is conducive to building good social relations. The standardised regression coefficient is 0.456, and better social relations are positively correlated with improvements in learning attitude. Harmonious and friendly social relations are conducive to improving international students’ attitude and enthusiasm for learning (see Figure 2).

### 3.3. Mediation Analysis

For Hypothesis 1, the mediation effect analysis involves three models, which are as follows:

Learning attitude = 2.798 + 0.286 × motivation to study abroad.

Language level = 2.622 + 0.376 × Motivation to study abroad.

Learning attitude = 2.096 + 0.185 × Motivation to study abroad + 0.267 × language level.

The motivation to study abroad (variable 2) has a significant positive impact on language level (variable 4). The influence coefficient is 0.376, indicating that when international students are intrinsically motivated to study abroad and their motivation is scientific and reasonable, they are enthusiastic about participating in teaching, communication and other aspects of learning and life and are more motivated to overcome language barriers. Improved language skills (variable 4) significantly positively impact learning attitude (variable 1). The influence coefficient of 0.267 indicates that when international students have a higher language level, they are better able to express their views, complete their homework and communicate, which is more conducive to forming positive learning attitudes, developing clear understanding and maintaining a high level of enthusiasm for learning. When language level (variable 4) is taken as the intermediary variable, the motivation to study abroad (variable 2) has a significant positive impact on learning attitude (variable 1), with an influence coefficient of 0.185. When international students have a clear and scientific understanding of the purpose of studying abroad and are highly motivated to do so, they are more likely to develop a good attitude towards learning and have a greater interest in learning and desire for knowledge.

**Figure 3:**
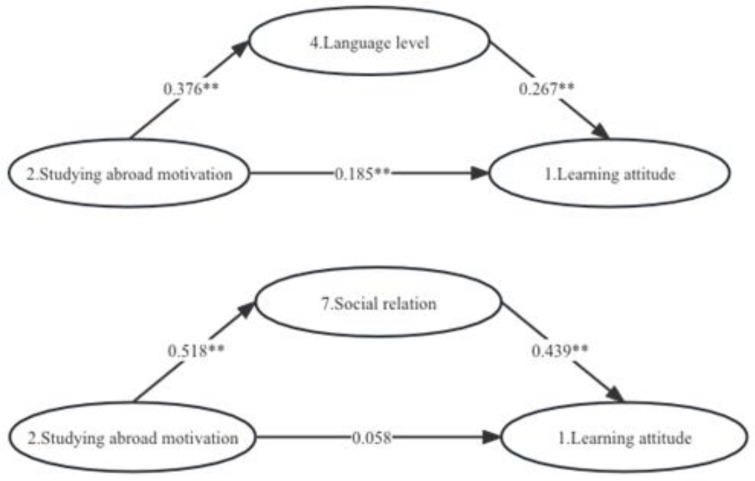
Pathway analysis. ** *p* < 0.01.

The mediating effect in this model is a partial mediating effect, with a total effect of 0.286, an intermediate effect of 0.101, a direct effect of 0.185 and an effect proportion of 35%. When language level (variable 4) is the mediating variable, 35% of the significant positive impact of study abroad motivation (variable 2) on learning attitude (variable 1) is manifested through the mediating effect. As the motivation to study abroad increases and the motivation composition becomes more reasonable, then the language level improves, enthusiasm for learning is increasingly cultivated and a more positive and correct learning attitude is established, improving the learning adaptability of international students in China (see Figure 3).

For Hypothesis 2, the mediation effect analysis involves three models as follows:

Learning attitude = 2.798 + 0.286 × motivation to study abroad.

Social connections = 2.057 + 0.518 × Motivation to study abroad.

Learning attitude = 1.895 + 0.058 × Motivation to study abroad + 0.439 × language level.

The motivation to study abroad (variable 2) has a significant positive impact on social relations (variable 7). The influence coefficient of 0.518 indicates that when international students have strong endogenous motivation and scientific and reasonable motivation to study abroad, they are more enthusiastic about participating in teaching, communication and other aspects of study and life. This enthusiasm will help establish good social relations through communication and cooperation. Social relationships (variable 7) have a significant positive impact on learning attitude (variable 1). The influence coefficient of 0.439 indicates that when international students have good social relationships, that is, communicating well, making friends, and participating in activities, they are conducive to developing a good attitude towards and interest in learning, adjusting negative emotions and cultivating a spirit of studying. When social relationships (variable 7) are the mediating variable, the motivation to study abroad (variable 2) has a significant positive impact on learning attitudes (variable 1), and the influence coefficient is 0.058. That is, when international students have a clear, scientific, and reasonable understanding of the purpose of studying abroad and a strong internal motivation, they are more likely to develop a positive attitude towards learning. Improving of social relations can contribute to cultivating enthusiasm for learning enthusiasm and effective learning behaviour.

The role of mediation in this model is full mediation, meaning that social relations completely mediate the influence of study abroad motivation on learning attitude. The total effect is 0.286, the intermediate effect is 0.058, the direct effect is 0.228, and the effect proportion is 100%, which is completely intermediary. This shows that the influence of study abroad motivation on learning attitude is fully reflected through social relationships. This reflects the overlap between the role of study abroad motivation actors and the social contact path in the learning adaptability of international students who come to China. The motivation of education actors, such as teachers, classmates, friends, and family, to recommend study abroad depends on the social networks between students and other subjects. Activity coordination and participation, information exchange and sharing, teaching and research management practices under social relations are the specific path of the orientation purpose of professional courses, Chinese culture, study, and employment. Therefore, the influence of study abroad motivation on learning attitude is fully realized through the specific practice path of social relations (see Figure 3).

## 4. Discussion

Seven influencing factors were found in the learning adaptability of international students in China: learning attitude, motivation to study abroad, learning ability, language level, learning environment, teaching management and social relations.

Learning attitude is a direct factor and key indicator reflecting learning adaptability. Language level is an important influencing factor on the adaptability of international students, and social relationships are an important influencing factor on the communication between international students and the environment. Of the seven variables, learning motivation has a significant positive effect on language level, with a coefficient of 0.183. Learning motivation has a significant positive effect on social relations, with a coefficient of 0.174. Learning ability has a significant positive effect on language level, with a coefficient of 0.2. Learning ability has a significant positive effect on social relations, with a coefficient of 0.254. Language level has a significant positive effect on learning attitude, with a coefficient of 0.152. The learning environment has a significant positive effect on social relations, with a coefficient of 0.29. Teaching management has a significant positive effect on language level, with a coefficient of 0.319. Teaching management has a significant positive effect on social relations, with a coefficient of 0.330. Social relationships have a significant positive impact on learning attitude, with a coefficient of 0.456.

Of the seven variables, the greatest influence on learning attitude is the expectation of course learning, with a standardization coefficient of 0.776. The recommendation of teachers, family members and friends to study in China significantly positively impacts learning motivation, and the coefficient is 0.756. The variable that has the second-greatest influence on learning ability is test performance, with a coefficient of 0.749. The most significant influence on language level is the ability to express ideas in Chinese, with a coefficient of 0.769. The greatest influence on the learning environment of international students is the accommodation environment, with a standardised regression coefficient of 0.797. The foreign students’ satisfaction with teaching has the greatest influence on teaching management, with a coefficient of 0.86. The number of Chinese friends of international students has the greatest influence on social relations, with a coefficient of 0.755.

When language level (variable 4) is used as the mediating variable, motivation to study abroad (variable 2) has a significant positive impact on learning attitude (variable 1), with an influence coefficient of 0.185 and an effect proportion of 35%, which is a partial mediator. When social relationships (variable 7) is used as the mediating variable, study abroad motivation (variable 2) has a significant positive impact on learning attitude (variable 1), with an influence coefficient of 0.058, which is completely mediating. This phenomenon can be attributed to the overlap of the main role of study abroad motivation and the contact object in social relationships.

The previous studies have also indicated the learning attitude, motivation to study abroad, learning ability, language level, learning environment, teaching management and social relations play a key role in the learning adaptability of international students, contextually. For example, Wei and Li [10] found that college students’ learning adaptability mainly includes five aspects: learning motivation, learning ability, environmental factors, teaching mode and learning attitude. Yang et al. [13] also pointed out that the main factors of college students’ learning adaptation include learning motivation, teaching mode, learning ability, learning attitude and environmental factors. Li et al. [16] found that there existed adjustment to Chinese culture and mental health issues among foreign students on Chinese university campuses during the COVID-19 pandemic from a collaborative ethnographic study perspective. In addition, Yu et al. [23] also highlighted the effects of academic adaptation on the depression of international students in China from a case study on South Asian students of a TCSOL teacher program.

### 4.1. Theoretical Implications

The findings of this study contribute to the literature on the factors that affect the learning adaptability of international students in China. The literature shows that the learning adaptability of international students is challenging. Cross-cultural adaptation theory in this study provides a solution to this challenge [11,16]. The findings show the learning adaptability of international students in China comprises seven variables: learning attitude, motivation to study abroad, learning ability, language level, learning environment, teaching management and social relations. Theoretically, it is crucial to discuss the factors that affect the learning adaptability of international students in China’s higher education context. This study focuses on the learning adaptability of foreign students in China. According to the cross-cultural adaptation theory, foreign students in China become involved with Chinese culture. The growth in the process of acculturation is reflected in three influencing factors, namely, communication, the environment, and personal characteristics, which also provide input for the design of this study [24,25,26].

### 4.2. Practical Implications

This study also highlighted that, in the learning process, especially in learning adaptation after exposure to a new culture, learners are affected by internal factors, such as basic knowledge and skills, learning motivation and learning self-efficacy. At the same time, learners’ adaptability is also restricted by external environmental factors, such as course content, course resources and teaching. Thus, studying the learning adaptability of international students requires comprehensively and systematically considering the influence of individual internal and external factors, understanding the learning adaptation behaviours, rules and problems encountered by, and improving and adjusting the variables of, external environmental factors and internal individuals to promote student learning adaptability after changing majors. Social communication, a major influencing factor in the theory of cross-cultural adaptation, is especially reflected in intercultural communication, which also provided ideas for this study [27].

### 4.3. Limitations and Future Research Directions

This study had several limitations. Future research should use a larger sample size to study the interactive effects and mediating roles of the multiple factors that influence the learning adaptative growth of international students. In addition, more interviews should be conducted to examine different stakeholders’ perspectives on promoting the learning adaptability of international students. Additionally, more comparative case studies should be included to explore different cultural backgrounds of the learning adaptability of international students.

## 5. Conclusions

This study aimed to explore what factors affect the learning adaptability of international students in China through structural equation modelling and mediation analysis. We found that seven influencing factors affect the learning adaptability of international students in China: learning attitude, motivation to study abroad, learning ability, language proficiency, learning environment, teaching management and social relations. As previous studies highlighted, Kim and Kim [9] argued that international students need to promote their learning adaption and learning ability in the host country. Shieh [21] also pointed out the effects of culture shock and cross-cultural adaptation play a key role in international students’ learning satisfaction, comprehensively. In the meantime, along with the model of acculturation process [9], we realize that international students should overcome the barriers of cultural differences. For instance, international students who are about to go abroad are advised to do as much as possible to prepare for the language and culture [28,29,30]. They need to find as many opportunities as possible to contact foreigners and ask them to introduce their cross-cultural experience, foreign living habits and so on. After going abroad, we should also boldly make local friends, and try our best to “do as the Romans do” in terms of lifestyle and code of conduct. It is an effective means of removing cultural barriers. Being desperate to “go your own way,” in the end, can only lead to becoming a “lonely person” [31].

## Figures and Tables

**Table 1:** Frequency analysis.

Item	Option	Frequency	Rate (%)	Accumulated Rate (%)
Gender	Male	2400	51.50	51.50
	Female	2260	48.50	100.00
Age	19–23 years old	2640	56.65	56.65
	Above 23	2020	43.35	100.00
Source of student	Asia	2030	43.56	43.56
	Africa	810	17.38	60.94
	Europe	650	13.95	74.89
	America	1170	25.11	100.00
Time length of learning Chinese (months)	1–6	290	6.22	6.22
	6–12	117	25.11	31.33
	12–24	185	39.70	71.03
	24–36	69	14.81	85.84
	More than 36	66	14.16	100.00
Chinese level	No level proof	72	15.45	15.45
	Level 3	27	5.79	21.24
	Level 4	43	9.23	30.47
	Level 5	138	29.61	60.09
	Level 6	186	39.91	100.00
Source of study abroad funding	Full scholarship exemption	206	44.21	44.21
	Half free scholarship	101	21.67	65.88
	Self-funded	127	27.25	93.13
	Other sources	32	6.87	100.00
First visit to China	Yes	377	80.90	80.90
	No	89	19.10	100.00
Experience of going abroad before coming to China	Never go abroad	287	61.59	61.59
	Have gone abroad before	179	38.41	100.00
Understanding of Beijing Normal University	Very unfamiliar	25	5.36	5.36
	Unfamiliar	60	12.88	18.24
	Average	136	29.18	47.42
	understand	164	35.19	82.62
	Very familiar	81	17.38	100.00
Experience of interacting with people from different cultural backgrounds	No	78	16.74	16.74
	Little	232	49.79	66.52
	Average	156	33.48	100.00
Have Chinese teachers to teach Chinese	Yes	336	72.10	72.10
	No	130	27.90	100.00
Foreign citizen of Chinese origin	Yes	157	33.69	33.69
	No	309	66.31	100.00
Religious belief	Christianity	111	23.82	23.82
	Islam	196	42.06	65.88
	Buddhism	106	22.75	88.63
	Other religion	29	6.22	94.85
	No	24	5.15	100.00
Accumulation		466	100.0	100.0

**Table 2:** Basic indicators.

Item	Size	Minimum	Maximum	Average	Standard Deviation	Median
Time length of learning Chinese	466	1.000	5.000	3.056	1.102	3.000
Chinese level	466	1.000	5.000	3.727	1.430	4.000
Understanding of Beijing Normal University	466	1.000	5.000	3.464	1.085	4.000

**Table 3:** Summary table of model regression coefficients.

X	→	Y	Non-Standardised Regression Coefficient	*SE*	*z* (CR Value)	*p*	Standardised Regression Coefficient
2. Studying abroad motivation	→	4. Language level	0.173	0.052	3.319	0.001	0.183
2. Studying abroad motivation	→	7. Social relation	0.160	0.042	3.791	0.000	0.174
3. Learning ability	→	4. Language level	0.199	0.056	3.525	0.000	0.200
3. Learning ability	→	7. Social relation	0.245	0.048	5.071	0.000	0.254
4. Language level	→	1. Learning attitude	0.148	0.051	2.892	0.004	0.152
5. Learning environment	→	7. Social relation	0.238	0.039	6.082	0.000	0.290
6. Teaching management	→	4. Language level	0.244	0.041	5.952	0.000	0.319
6. Teaching management	→	7. Social relation	0.245	0.034	7.277	0.000	0.330
7. Social relation	→	1. Learning attitude	0.455	0.058	7.890	0.000	0.456

Note: → represents the regression influence relationship or measurement relationship.

## Data Availability

The datasets generated and/or analysed during the current study are available from the corresponding author upon reasonable request.
